# *Portulaca oleracea* L. Repairs the Skin Barrier and Alleviates Atopic Dermatitis by Targeting Lipid Metabolism to Regulate the JAK1/STAT3 Signaling Pathway and Downregulate Th2 Inflammatory Cytokines

**DOI:** 10.3390/biomedicines14092006

**Published:** 2026-09-07

**Authors:** Jiangyan Yong, Kun Yang, Yiman Ge, Guining Luo, Yaohui Zhu, Lihua Luo, Jiaqi Li, Xinyi Xiang, Weijun Ding, Yimei Hu

**Affiliations:** 1 School of Basic Medical Sciences, Chengdu University of Traditional Chinese Medicine, Chengdu 611137, China; 2 Hospital of Chengdu University of Traditional Chinese Medicine, Chengdu 610072, China; 3 Chengdu University of Traditional Chinese Medicine, Chengdu 611137, China

**Keywords:** *Portulaca oleracea* L., atopic dermatitis, JAK1/STAT3 signaling, skin barrier, metabolomics

## Abstract

**Background:*** Portulaca oleracea* L. (POL) has anti-inflammatory and immunomodulatory activities, but its mechanism against atopic dermatitis (AD) remains incompletely understood. **Methods:** 2,4-dinitrofluorobenzene (DNFB)-induced AD mice were treated orally with POL or dexamethasone for 14 days. We evaluated skin lesions, scratchizng, serum IgE, histopathology, inflammatory cytokines, skin-barrier genes, JAK/STAT phosphorylation, and fecal metabolites. **Results:** POL alleviated skin lesions and pruritus, reduced serum IgE concentrations, attenuated epidermal hyperplasia and inflammatory-cell and mast-cell infiltration, and restored keratinocyte architecture. It decreased IL-4, IL-13, and IL-31 expression, increased filaggrin and loricrin expression, and inhibited JAK1, STAT1, and STAT3 phosphorylation. Untargeted metabolomics showed that POL mainly regulated unsaturated fatty acid and steroid hormone biosynthesis and restored levels of anti-inflammatory and antiallergic metabolites, including (±)18-HEPE, docosahexaenoyl ethanolamide, and dehydroepiandrosterone. **Conclusions:** These findings indicate that POL ameliorates AD by suppressing Th2 inflammation through JAK1/STAT3 signaling, correcting lipid-metabolic disturbances, and restoring skin barrier integrity.

## 1. Introduction

Atopic dermatitis (AD) is a chronic inflammatory dermatosis characterized by dermal papules, edema, crusting, scaling, recurrent eczema, and significant pruritus [1]. The global incidence of AD is 327.91 per 100,000 person-years, alongside a prevalence rate of 2.62%, which affects approximately 204 million individuals worldwide [2,3]. Furthermore, AD is strongly correlated with the development of food allergies, allergic rhinitis and asthma, collectively identified as the “atopic march”. The principal mechanisms underlying AD pathogenesis include immune dysregulation, compromised skin barrier integrity, and dysbiosis. Specifically, an imbalance in the Th2 inflammatory process is a key aspect of AD, wherein Th2 cells secrete proinflammatory cytokines such as interleukin (IL)-4, IL-13, and IL-31. These cytokines activate the Janus kinase (JAK) signaling pathway to exacerbate inflammation and pruritus [1,4] and downregulate filaggrin (FLG) expression in the skin barrier, promoting allergen penetration [5,6]. This impairment of the skin barrier facilitates microbial dysbiosis, characterized by the colonization of Staphylococcus aureus [7].

Conventional interventions for AD primarily aim to suppress cutaneous inflammation and restore barrier function, with glucocorticoids serving as the first-line treatment for inflammation and pruritus in mild to moderate AD [1]. However, systemic immunosuppressants such as glucocorticoids, cyclosporine and methotrexate fail to address the fundamental immune dysregulation associated with AD, leading to frequent recurrences and increasing risks of severe adverse effects [1,6]. Alternatively, targeting pivotal Th2 cytokines represents a promising paradigm. Biologic agents, such as dupilumab and tralokinumab, and JAK inhibitors, such as abrocitinib and upadacitinib, have been approved for moderate-to-severe AD and have shown considerable efficacy [8,9]. Nevertheless, these innovative therapies are accompanied by significant risks, including susceptibility to skin infections, exacerbation of asthma, and increased thromboembolism and malignancy [10]. Thus, the development of safe and effective therapeutics for AD is urgently needed.

*Portulaca oleracea* L. (POL) is an edible and medicinal herb that is extensively used in traditional Chinese medicine (TCM). Documented by Li Shizhen in the Ming Dynasty, POL was historically used for detoxification, anti-swelling, and alleviation of diarrhea [11]. Recent pharmaceutical research has revealed its extensive effects, including anti-inflammatory, antibacterial, antitumor, and antioxidant activities [11,12], as well as neuroprotective and immunomodulatory activities [13,14]. Chemical assays have shown that POL contains a variety of bioactive compounds, including alkaloids, flavonoids, fatty acids, catecholamines, phenolic acids, anthocyanins, lignans, and terpenoids [12]. Notably, POL is rich in ω-3/ω-6 fatty acids, specifically α-linolenic acid, γ-linolenic acid, and linoleic acid, which are rarely abundant in terrestrial plants [15,16]. ω-3 fatty acids and their derivatives are recognized for their significant anti-inflammatory properties [17]. Increased concentrations of putrescine and sphingomyelin have been documented in the epidermis of individuals diagnosed with AD [18,19]. A significant reduction in ω-3/ω-6 polyunsaturated fatty acids (PUFAs) has been detected in both the skin and serum of patients with AD, specifically eicosapentaenoic acid (EPA) and docosahexaenoic acid (DHA) [20,21].

Furthermore, the levels of tryptophan and its metabolic derivatives, which are instrumental in immune modulation, are markedly decreased in AD patients [22]. However, the molecular mechanism through which POL affects AD remains unclear. Accordingly, this study was undertaken to systematically evaluate the therapeutic efficacy of POL in a DNFB-induced murine AD model and to dissect its mechanistic basis. In contrast to prior investigations that predominantly emphasized broad anti-inflammatory actions, the present work contributes original evidence in two key respects: first, by assessing whether POL attenuates Th2-driven cytokine production (IL-4, IL-13, and IL-31) via targeted suppression of JAK1/STAT3 phosphorylation, and second, by applying untargeted fecal metabolomics to delineate, for the first time, the restorative effects of POL on AD-associated lipid dysmetabolism, specifically its effects on unsaturated fatty acid and steroid hormone biosynthetic pathways. Collectively, the integration of pharmacodynamic, molecular, and metabolomic approaches offers a multidimensional mechanistic framework that supports POL as a potential therapeutic candidate for AD.

## 2. Materials and Methods

### 2.1. Preparation of POL Solution

The aqueous extract of POL was prepared according to the protocol described by Wang et al. [23]. The aerial parts of POL were collected, air-dried, and ground into coarse powder. Dried POL was obtained from the Affiliated Hospital of the Chengdu University of TCM (Chengdu, China) and was identified by Dr. Hongping Chen at the Chengdu University of TCM. Dried POL was obtained from the Affiliated Hospital of the Chengdu University of TCM (Chengdu, China) and was identified by Dr. Hongping Chen at the Chengdu University of TCM. A total of 15.17 g of POL was immersed in 200 mL of distilled water for 30 min and decocted for 30 min. The procedure was repeated twice. The three decocted solutions were combined, concentrated to a final volume of 50 mL (*w*/*v*: 303.4 mg/mL) using a rotary evaporator, filtered through a 0.22-μm filter, and stored at 4 °C until use. Aqueous extraction was chosen to mimic the traditional decoction mode of POL, as this procedure preserves the integrative phytochemical profile and colloidal stability that may enhance the solubilization of its diverse constituents.

### 2.2. Liquid Chromatography–Mass Spectrometry (LC–MS) Analysis of POL Solutions

A total of 100 μL of the POL solution was mixed with 100 μL of 70% methanol containing 2-chloro-L-phenylalanine as the internal standard (final concentration: 100 ng/mL). The resulting mixture was vortex-mixed for 15 min at 12,000 revolutions per minute at a controlled temperature of 4 °C, followed by centrifugation for 3 min. The supernatant obtained from this process was filtered through a 0.22 μm microporous membrane and subsequently stored in an injection vial designated for LC–MS analysis. Chromatographic separation was performed on an ACQUITY UPLC HSS T3 column (2.1 × 100 mm, 1.8 μm) at a flow rate of 0.40 mL/min. The mobile phase consisted of 0.1% formic acid in water (solvent A) and 0.1% formic acid in acetonitrile (solvent B). The gradient program was: 0–5 min, 95% A → 35% A; 5–6 min, 35% A → 1% A; held at 1% A for 1.5 min. Data acquisition was performed in information-dependent acquisition (IDA) mode using Analyst TF 1.7.1 Software (Sciex, Concord, ON, Canada). This sample preparation method was used to assess the constituents present in the POL aqueous extract analytically. Mass spectrometric detection was performed on a Triple TOF 5600+ system (Sciex) with a DuoSpray™ ion source in both positive and negative electrospray ionization modes. The source parameters were set as follows: ion source gas 1 (GAS1), 50 psi; ion source gas 2 (GAS2), 60 psi; curtain gas (CUR), 35 psi; temperature (TEM), 550 °C; declustering potential (DP), +80 V in positive mode and −80 V in negative mode; and ion spray voltage floating (ISVF), +5500 V in positive mode and −4500 V in negative mode. The TOF MS scan parameters were: mass range, *m*/*z* 50–1250; accumulation time, 200 ms; and dynamic background subtraction, on. Product ion scan parameters were: mass range, *m*/*z* 50–1250; accumulation time, 40 ms; collision energy, +30 V in positive mode and −30 V in negative mode; collision energy spread, 15; resolution, UNIT; charge state, 1 to 1; intensity threshold, 100 cps; exclude isotopes within 4 Da; mass tolerance, 50 mDa; and maximum number of candidate ions monitored per cycle, 12.

### 2.3. AD Model, POL Treatment and Sample Collection

C57BL/6J male mice, aged 8 weeks and weighing between 18 and 20 g, were procured from Beijing Sipeifu Biological Science Co., Ltd. (Beijing, China). Mice were housed under controlled conditions: a room temperature of 25 ± 2 °C, a relative humidity of 40–60%, and a 12-h light/dark cycle. They had free access to food and water.

After the acclimation period, the mice were randomly allocated to six experimental groups: normal, model, POL-L (1.5 g/kg), POL-M (3 g/kg), POL-H (6 g/kg), and Dex (1 mg/kg, the positive control). Dexamethasone (Dex; Biosharp, HeFei China) was dissolved in a saline solution. The dorsal fur of the mice was removed within an area of 2.5 × 2.5 cm, and sensitization was conducted via a 2% 2,4-dinitrofluorobenzene (DNFB; Sigma Aldrich, St. Louis, MO, USA) acetone solution (where the ratio of acetone to olive oil was 3:1, utilizing a volume of 50 μL) on days 0 and 3, followed by the administration of a 0.5% DNFB solution on days 6, 9, 12, 15, and 18.

Mice in the normal and model groups only received the acetone solvent. On day 7, the mice received either POL extract or Dex via gavage for 14 consecutive days, whereas the normal group received saline. We systematically monitored parameters including diet, activity levels, and the extent of skin damage. On day 22, the severity of the skin lesions was quantitatively assessed via a scoring system (ranging from 0–12) that evaluated erythema, scarring, edema, and desquamation before euthanasia, at which point blood samples were also obtained.

### 2.4. Histopathological Analysis

Mouse dermal tissue samples were fixed in 4% paraformaldehyde, subsequently embedded in paraffin, and cut into 5 μm sections. The sections were stained with hematoxylin and eosin (H&E) and toluidine blue. Photomicrographs were obtained via a Leica DM6B microscope, and three randomly selected fields per specimen were evaluated to quantify the infiltrating mast cells within the dermal layer.

### 2.5. Scanning Electron Microscope Assay

Skin tissue samples were rinsed with physiological saline and subsequently fixed in a 3% glutaraldehyde solution. After thorough rinsing, the samples were fixed in a 1% osmium tetroxide solution for one hour, then dehydrated in a series of graded ethanol solutions. The dehydrated samples were mounted on aluminum stubs using conductive adhesive and subsequently coated with a thin metallic layer via an ion sputtering apparatus. Finally, morphological images were acquired via a scanning electron microscope (JSM-IT700HR, JEOL, Tokyo, Japan).

### 2.6. Enzyme-Linked Immunosorbent Assay (ELISA)

Blood samples were obtained from the mice after euthanasia, and the serum was isolated via centrifugation at 2000 rpm for 10 min and subsequently stored at 4 °C. IgE levels were quantified using a Mouse IgE (Immunoglobulin E) ELISA Kit (Elabscience, HanWu, China) according to the manufacturer’s instructions. Optical density (OD) measurements were recorded at a wavelength of 450 nm via a microplate reader, and IgE concentrations were calculated from the standard curve.

### 2.7. Real-Time PCR

Total RNA was extracted from skin tissue using an Animal Total RNA Isolation Kit (Foregene, ChengDu, China) and reverse transcribed into cDNA with a HiScript III 1st Strand cDNA Synthesis Kit (Vazyme, Nanjing, China). PCR amplification was performed using a ChamQ SYBR qPCR Master Mix Kit (Vazyme, Nanjing, China). Relative target gene expression fold changes were calculated via the 2^−ΔΔCt^ method. The primer sequences are listed in Table 1.

### 2.8. Untargeted Metabolomics

Untargeted metabolomics utilizing LC–MS technology was performed, encompassing metabolite extraction, LC–MS detection, and subsequent data analysis. The fecal samples (100 mg) were homogenized in liquid nitrogen, reconstituted in prechilled 80% methanol, incubated on ice for 5 min, and centrifuged at 15,000× *g* at 4 °C for 20 min. The resulting supernatant was diluted to a concentration of 53% methanol and subsequently injected into the LC–MS system.

The data were processed using Compound Discoverer 3.1 (Thermo Fisher Scientific, Waltham, MA, USA) and normalized before multivariate statistical analyses, including principal component analysis (PCA), partial least squares discriminant analysis (PLS-DA), and orthogonal partial least squares discrimination analysis (OPLS-DA). Differentially expressed metabolites were identified based on criteria of log2(fold change) > 0.263 or <−0.263 and *p* < 0.05, and their functional roles and metabolic pathways were examined using the Kyoto Encyclopedia of Genes and Genomes (KEGG) database.

### 2.9. Western Blot

Skin tissues were homogenized in RIPA lysis buffer (Beyotime, ShangHai, China) supplemented with protease and phosphatase inhibitors (Roche, Basel, Switzerland). Lysates were centrifuged at 12,000× *g* for 15 min at 4 °C, and protein concentrations were determined using a BCA assay kit (Thermo Fisher Scientific, Waltham, MA, USA). Equal amounts of protein (30 μg) were resolved by 10% SDS-PAGE and transferred onto PVDF membranes (Millipore, Burlington, MA, USA). Membranes were blocked with 5% non-fat milk in TBST for 1 h at room temperature and probed with primary antibodies overnight at 4 °C, followed by incubation with an HRP-conjugated secondary antibody for 1 h at room temperature. Antibody information is summarized in Table 2. Protein bands were visualized using an enhanced chemiluminescence substrate (Millipore, Burlington, MA, USA) and imaged with a ChemiDoc system (Bio-Rad, Hercules, CA, USA). Band intensities were quantified using ImageJ 1.54p software (NIH, Bethesda, MD, USA), with target protein levels normalized to β-actin and phosphorylated proteins normalized to their respective total protein levels.

### 2.10. Statistical Analysis

Statistical analysis was performed using one-way analysis of variance (ANOVA) followed by Tukey’s post-hoc multiple comparison test for all multi-group comparisons, using GraphPad Prism software (version 10). Findings are presented as the mean ± standard error of the mean (SEM), with *p* < 0.05 considered statistically significant.

## 3. Results

### 3.1. POL Was Enriched in Compounds Including Unsaturated Fatty Acids

To investigate the therapeutic basis of POL for AD, compositional analysis of the POL aqueous extract was initially performed employing LC–MS. The results demonstrated that the POL aqueous extract predominantly contained flavonoids, lipids, phenolic acids, terpenoids, lignans and coumarins, and organic acids, among other compounds (Table 3). Notably, the POL aqueous extract exhibited relatively abundant signals corresponding to ω-3 and ω-6 unsaturated fatty acids, including γ-linolenic acid, eicosapentaenoic acid (EPA), and linoelaidic acid, based on their chromatographic peak intensities in the untargeted LC-MS profile. These unsaturated fatty acids and their metabolites are known to possess potent anti-inflammatory properties, suggesting their potential relevance to the mechanism through which POL ameliorates AD through suppression of inflammatory responses and restoration of skin barrier function.

### 3.2. General Conditions

**Figure 1:**
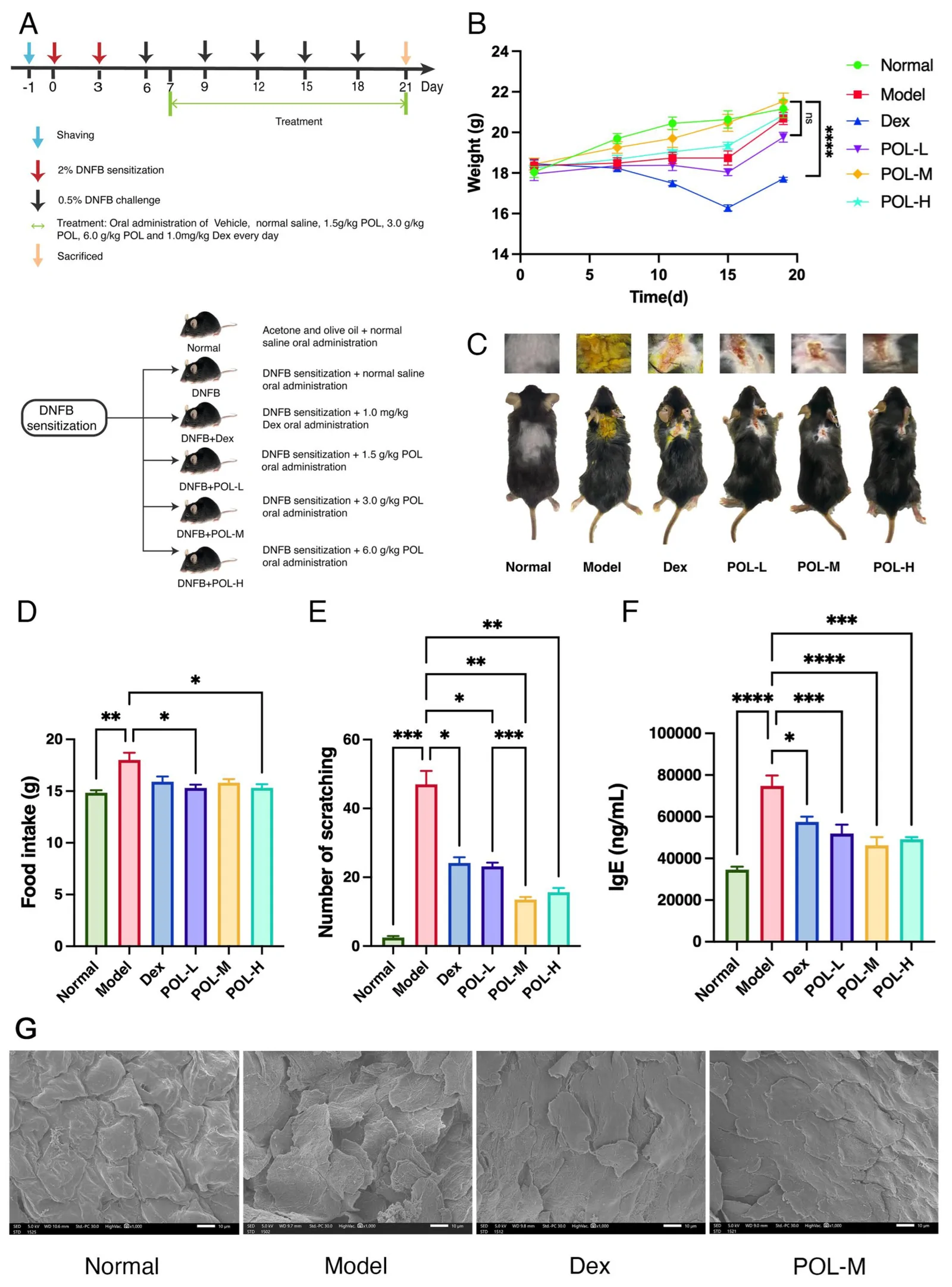
Effects of POL on clinical symptoms in DNFB-induced AD mice. (**A**) Construction process of the AD model and drug administration method. POL-L, 1.5 g/kg; POL-M, 3 g/kg; POL-H, 6 g/kg; Dex, 1.0 mg/kg. n = 6/group. (**B**) Body weight of mice on days 1, 7, 11, 15 and 19. Body weight was compared across groups at each time point using one-way ANOVA with Tukey’s multiple comparisons test. (**C**) Representative images of cutaneous lesions collected on day 22 (the day of sacrifice). (**D**) Daily food intake per cage, with 6 mice per cage. (**E**) The number of scratches per 10 min on day 22. (**F**) Serum IgE level (n = 5). (**G**) Representative cutaneous lesions of mice obtained using a scanning electron microscope (1000×). Data are expressed as mean ± SEM. * *p* < 0.05, ** *p* < 0.01, *** *p* < 0.001, **** *p* < 0.0001.

Compared with the normal group, the model group presented marked cutaneous lesions and a marked increase in food consumption, whereas the Dex group exhibited a significant reduction in body weight. The experimental model was established in accordance with the methodology delineated in Figure 1A. No statistically significant differences in body weight were observed among the normal, model, and POL groups (*p* > 0.05); however, the Dex group presented a significant reduction in body weight during the later stages of the treatment regimen (19 days) (Figure 1B, *p* < 0.05). Food consumption did not differ significantly among the normal, Dex, and POL groups throughout the treatment period (*p* > 0.05); conversely, the Model group showed a significant increase in food intake (Figure 1D, *p* < 0.05). These findings suggest that the administration of oral dexamethasone for the treatment of AD is associated with pronounced adverse effects.

### 3.3. POL Markedly Alleviated Major AD-like Symptoms

The Model cohort demonstrated substantial cutaneous impairment, whereas the POL intervention effectively mitigated AD-like clinical manifestations, with the POL-M cohort exhibiting the most pronounced therapeutic efficacy. As shown in Figure 1C, the Model cohort exhibited severe damage, including inflammation, ulceration, and skin hyperplasia. Both the Dex and POL interventions alleviated cutaneous lesions and inflammation. Compared with the Model cohort, the POL cohort presented significantly diminished clinical manifestations, decreased skin crusting, erythema, and edema (Figure 1C). Moreover, the scratching frequency was significantly greater in the Model cohort (*p* < 0.05), but markedly lower following Dex and POL intervention (Figure 1E, *p* < 0.05). Serum IgE concentrations, which were significantly elevated in the Model cohort (*p* < 0.05), also declined after Dex and POL treatment (Figure 1F, *p* < 0.05), with the POL-M cohort showing the lowest scratching frequency and serum IgE concentrations. These findings suggest that the POL-M cohort (3 g/kg) had the most efficacious therapeutic outcome, indicating that this optimal dosage was suitable for subsequent mechanistic investigations. Electron microscopy revealed that keratinocytes in normal skin were well-organized with intact intracellular junctions, whereas keratinocytes in the Model cohort exhibited marked structural damage and desquamation. Both Dex and POL interventions attenuated this impairment, mitigating DNFB-induced AD symptoms (Figure 1G).

### 3.4. POL Alleviated Inflammatory Cell Infiltration and Epidermal Hyperplasia

**Figure 2:**
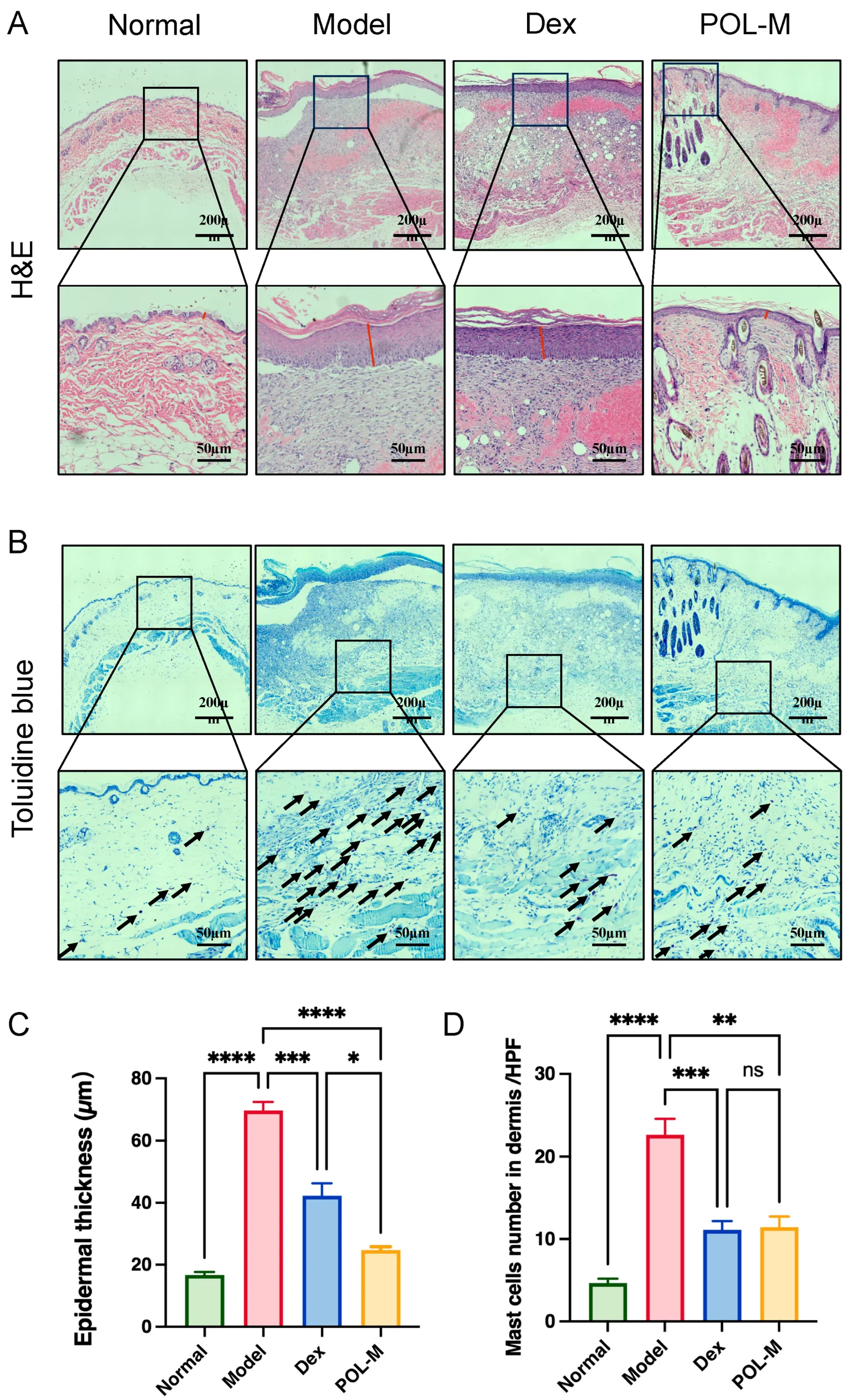
Histopathological analysis of the effects of POL on AD mice. (**A**) Representative cutaneous lesion and POL repair images are shown after hematoxylin and eosin (H&E) staining. Red line represents the epidermis (scale bars, 50 μm). (**B**) Representative microphotographs of cutaneous lesions stained with toluidine blue. Arrows indicate mast cells (scale bars, 50 μm). (**C**) Comparison of epidermal thickness. (**D**) Comparison of infiltrated mast cell numbers in dermal layers. n = 3/group. POL-M, 3 g/kg; Dex, 1.0 mg/kg. Data are expressed as mean ± SEM. * *p* < 0.05, ** *p* < 0.01, *** *p* < 0.001, **** *p* < 0.0001.

To further evaluate the therapeutic potential of POL in AD, histopathological analyses were conducted using HE and toluidine blue staining (Figure 2). HE staining showed that the Normal cohort had a well-preserved epidermal architecture, with normal thickness and no inflammatory infiltration. In contrast, the Model cohort exhibited pronounced epidermal hyperplasia (*p* < 0.05), accompanied by dense inflammatory cell infiltration and, in some instances, epidermal–dermal separation. Notably, treatment with either Dex or POL-M markedly reduced epidermal thickness and significantly mitigated inflammatory infiltration (Figure 2A,C; *p* < 0.05).

Given the pivotal role of mast cells as effector cells in AD pathogenesis, toluidine blue staining was employed to visualize metachromatic mast cells, which are readily identified by their deep purple staining (Figure 2B). In the Normal cohort, mast cells were sparsely distributed within the dermis, whereas the Model cohort demonstrated a significant elevation in mast cell density (Figure 2D; *p* < 0.05). Importantly, both Dex and POL-M interventions substantially reduced dermal mast cell infiltration (*p* < 0.05), further underscoring their anti-inflammatory efficacy.

### 3.5. POL Inhibited Inflammatory Cytokines and Repaired the Skin Barrier

**Figure 3:**
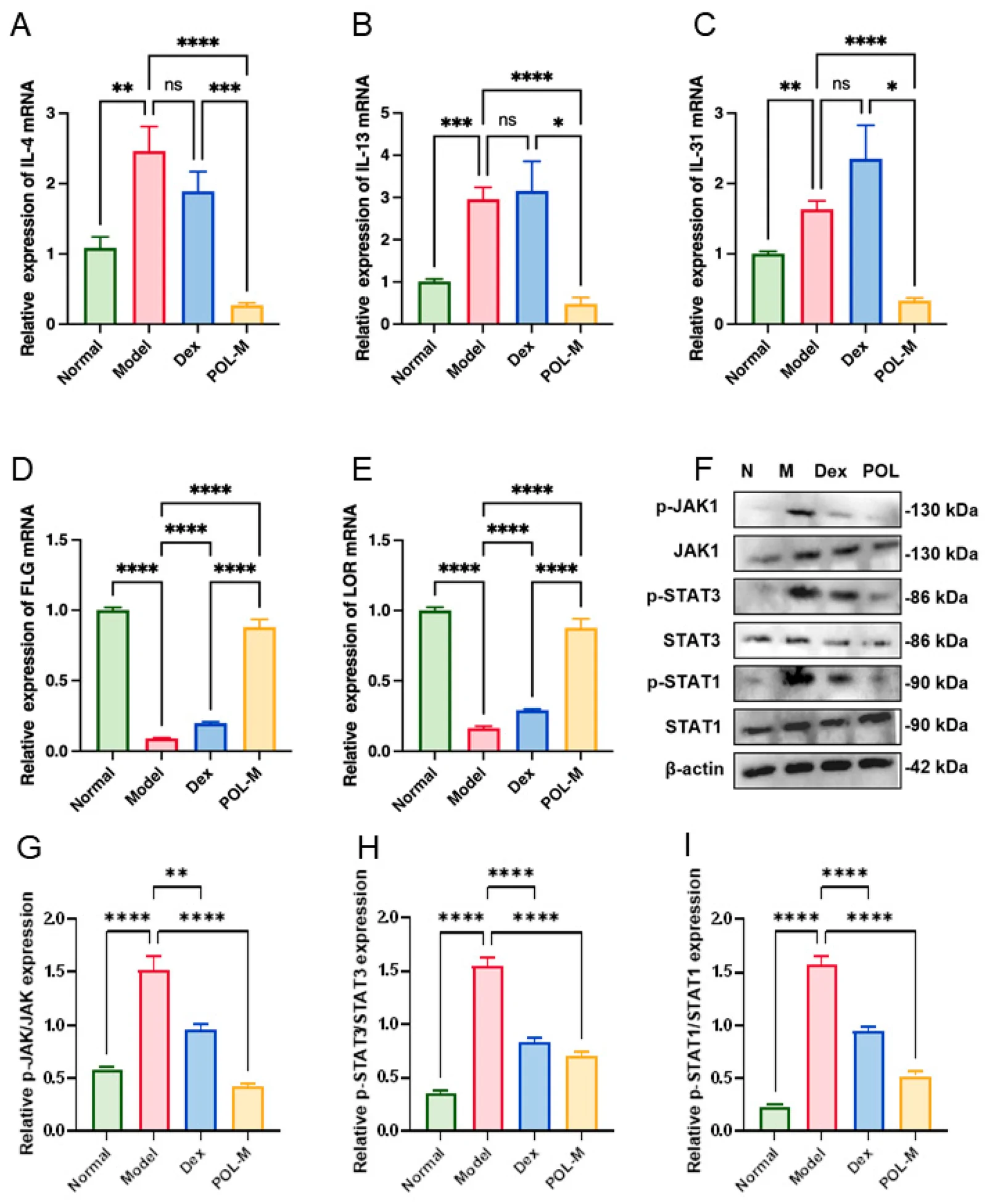
POL ameliorated skin inflammation and barrier function in AD mice through the JAK1-STAT3 pathway. (**A**–**C**) Relative mRNA expression levels of inflammatory cytokines IL-4, IL-13, and IL-31. (**D**,**E**) Relative mRNA expression levels of skin barrier-related genes FLG and LOR. Total RNA was isolated from the skin tissues of AD mice. (**F**–**I**) Protein expression of p-JAK1, p-STAT1, and p-STAT3 in skin tissues from AD mice. n = 3/group. POL-M, 3 g/kg; Dex, 1.0 mg/kg. Data are expressed as mean ± SEM. * *p* < 0.05, ** *p* < 0.01, *** *p* < 0.001, **** *p* < 0.0001.

To investigate how POL ameliorates AD, we examined mRNA expression levels of AD-related proinflammatory cytokines (IL-4, IL-13, and IL-31) and genes associated with barrier integrity (FLG and LOR) in skin tissue. The results demonstrated that POL substantially inhibited the DNFB-induced upregulation of IL-4, IL-13, and IL-31 mRNA (*p* < 0.05), whereas Dex failed to reduce the expression of these cytokines (Figure 3A–C). Importantly, POL also counteracted the DNFB-induced decrease in FLG and loricrin (LOR) mRNA levels (Figure 3D–E, *p* < 0.05), thus restoring skin barrier integrity. These observations imply that the therapeutic effects of POL on AD are closely associated with its ability to attenuate Th2 cell-mediated inflammatory responses and restore skin barrier integrity.

### 3.6. POL Suppressed Overactivation of the JAK1/STAT3 Pathway in the Skin

To examine the prospective signaling pathways implicated in the ameliorative effects of POL on AD, we scrutinized the activation of JAK1, STAT1, and STAT3 in dermal tissue. As shown in Figure 3F, the phosphorylation levels of p-JAK1, p-STAT1, and p-STAT3 were significantly increased in the dermal samples of the model group (*p* < 0.05). Conversely, the POL intervention considerably diminished the phosphorylation of p-JAK1, p-STAT1, and p-STAT3 (*p* < 0.05), whereas the Dex intervention only inhibited p-JAK1 and p-STAT3 phosphorylation. These observations suggest that POL’s mitigation of AD dermal lesions and inflammatory responses is closely linked to the JAK1/STAT3 signaling pathway.

### 3.7. POL Altered the Metabolic Profile of AD Mice

**Figure 4:**
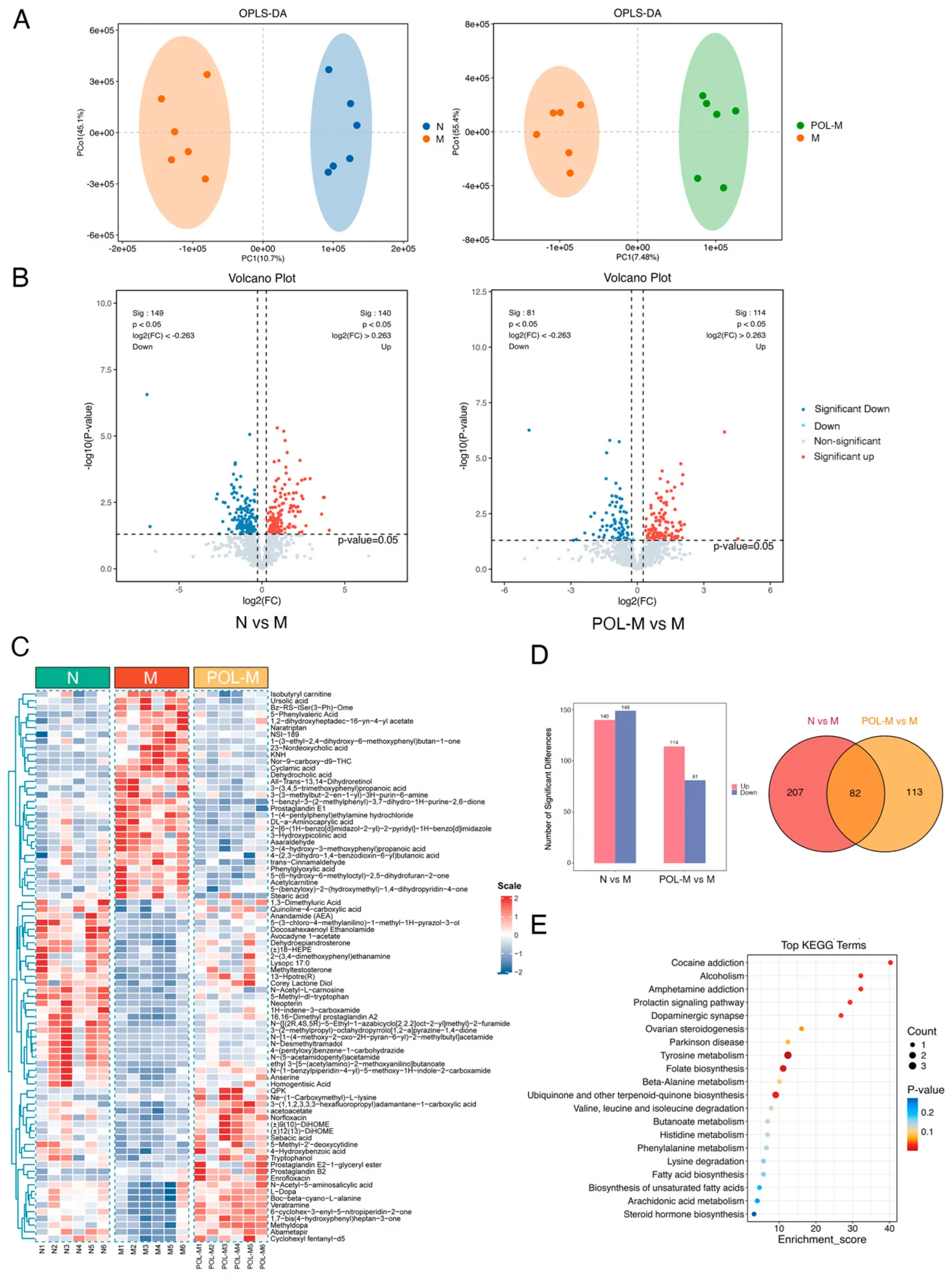
Characteristic metabolic profile of POL in the treatment of AD. (**A**) The OPLS-DA score plot of the metabolic data. (**B**) The volcano plot of differential metabolites. The horizontal axis represents the fold change of metabolites between groups, while the vertical axis indicates the *p*-value from the *t*-test. Differentially abundant metabolites were identified based on log2(FC) > 0.263 or <−0.263 and *p* < 0.05. (**C**) Heatmap analysis of differentially abundant metabolites. The horizontal axis represents the Normal, Model, and POL-M groups. The vertical axis indicates differentially expressed metabolites identified through intergroup comparison. Red denotes upregulated metabolites, while blue indicates downregulated metabolites. (**D**) Number of significant differences and Venn diagram. (**E**) KEGG analysis of differentially abundant metabolites. Differentially abundant metabolites in both the Normal and POL-M group were identified through comparison with the Model group. n = 6/group. POL-M, 3 g/kg; Dex, 1.0 mg/kg.

To investigate the effects of POL on metabolic profile alterations in AD, LC–MS analysis was performed on fecal samples from mice, which identified 1340 metabolites (970 in positive ion mode and 370 in negative ion mode). Using an OPLS-DA model, significant metabolic differences were identified between the normal and model groups as well as between the POL and model groups, as shown by the clear separation in the OPLS-DA score plot (Figure 4A). Differentially abundant metabolites were identified based on log2(FC) > 0.263 or <−0.263 and *p* < 0.05, revealing 289 altered metabolites in the normal group (140 upregulated and 149 downregulated) and 195 in the POL group (114 upregulated and 81 downregulated) compared with the model group. The volcano plots in Figure 4B highlight the distribution of these changes, with upregulated and downregulated metabolites represented as red and blue dots, respectively. To pinpoint metabolites that POL specifically influences in the treatment of AD, a Venn diagram was used to identify 82 overlapping differentially abundant metabolites between the normal vs. model and POL vs. model comparisons (Figure 4D).

POL predominantly modulated fatty acyls, steroid lipids, organic acids and their derivatives, benzenoids, and organoheterocyclic compounds, with fatty acyls constituting the predominant class (Figure 4C). In the Model cohort, stearic acid, acetylcarnitine, isobutyryl carnitine, and 5-phenylvaleric acid were markedly upregulated, and anti-inflammatory unsaturated fatty acids such as (±)18-HEPE, docosahexaenoyl ethanolamide, (±)12(13)-DiHOME, (±)9(10)-DiHOME, avocadyne 1-acetate, 13-HpOTrE(R), acetoacetate, and sebacic acid were downregulated, and POL intervention reversed these alterations by decreasing four lipid metabolites and increasing eight anti-inflammatory fatty acids. Among arachidonic acid metabolites in the Model cohort, there were elevated prostaglandin E1 and prostaglandin B2 levels, accompanied by reductions in anandamide, 16,16-dimethyl prostaglandin A2, and prostaglandin E2-1-glyceryl ester; POL intervention reversed these alterations by upregulating anandamide and associated compounds while decreasing prostaglandin E1. Furthermore, anti-inflammatory substances such as dehydroepiandrosterone (DHEA), 1,3-dimethyluric acid, and quinoline-4-carboxylic acid, in addition to antibacterial agents, including norfloxacin, enrofloxacin, and 4-hydroxybenzoic acid, along with amino acid metabolites such as 5-methyl-dl-tryptophan, N-acetyl-L-carnitine, and carnosine, were significantly downregulated in the Model cohort but were restored by the POL intervention, as shown in Table 4.

KEGG enrichment analysis revealed that the differentially abundant metabolites resulting from POL treatment were predominantly involved in lipid metabolic pathways. The top 20 enriched metabolic pathways included fatty acid biosynthesis, unsaturated fatty acid biosynthesis, steroid hormone biosynthesis, butanoate metabolism, arachidonic acid metabolism, ubiquinone and other terpenoid-quinone metabolism, histidine metabolism, tyrosine metabolism, phenylalanine metabolism, dopaminergic synapses, and folate biosynthesis (Figure 4E).

## 4. Discussion

This investigation demonstrated the therapeutic effects and underlying mechanisms of POL aqueous extract in ameliorating DNFB-induced AD in mice. Our results revealed that POL mitigated AD manifestations, including dermal inflammation, erythema, edema, ulceration, and increased epidermal thickness. It also significantly reduced pruritic behavior and IgE levels while curtailing infiltration of inflammatory leukocytes and mast cells into the skin. Moreover, POL reversed DNFB-induced perturbations to keratinocyte architecture and restored cutaneous barrier function.

AD is a persistent, recurrent inflammatory dermatosis characterized by Th2 cell dysfunction and compromised cutaneous barrier integrity. Patients with AD show dysregulated Th1/Th2 immune responses, with activated Th2 cells eliciting type 2 inflammatory pathways. Cytokines such as IL-4, IL-5, IL-13, and IL-31 are overexpressed during type 2 inflammation [24]. Among these, IL-4 and IL-13 are pivotal to Th2 responses, facilitating the differentiation of Th0 cells into Th2 cells and augmenting IgE synthesis, which is essential for hypersensitivity reactions. Th2 cytokines modulate mast cell and eosinophil function, resulting in inflammation and tissue injury in AD. IL-31 is linked to pruritus, intensifying scratching behavior and further undermining the cutaneous barrier [25]. This investigation revealed that POL significantly reduced mRNA levels of Th2-associated cytokines, including IL-4, IL-13, and IL-31, thereby suppressing Th2-type inflammatory responses.

The JAK/STAT signaling cascade is a crucial immune regulatory pathway involved in modulating Th2 inflammation. To further elucidate POL’s anti-inflammatory and immunoregulatory mechanisms, we evaluated its effects on the JAK/STAT signaling cascade. The JAK1/STAT3 pathway, activated by Th2 cytokines such as IL-4, IL-13, and IL-31, facilitates immune dysregulation and inflammation in AD [26]. Furthermore, JAK1/STAT3 are implicated in maintaining epidermal barrier integrity and peripheral nerves associated with pruritus transduction [27,28]. Activation of the JAK/STAT pathway also contributes to dysregulated lipid metabolism in AD lesional skin [29]. JAK inhibitors, including tofacitinib and ruxolitinib, have shown efficacy in managing AD by targeting the JAK/STAT pathway to mitigate symptoms and restore barrier function [30]. Our research showed that POL inhibited phosphorylation of p-JAK1, p-STAT1, and p-STAT3, thereby mitigating Th2-type inflammatory responses. AD is further characterized by pervasive skin barrier dysfunction, manifesting as the downregulation of barrier-associated molecules such as FLG, LOR, and involucrin (IVL), which constitute critical components of lesional skin in AD [31]. POL administration significantly increased the mRNA expression levels of FLG and LOR in cutaneous tissue. Previous investigations suggest that interleukins IL-4 and IL-13 can diminish FLG, LOR, and IVL expression through activation of STAT3 or STAT6 [32,33,34]. Importantly, the Th2-polarized inflammatory milieu present in AD exerts a more pronounced influence on FLG downregulation than loss-of-function mutations within the FLG gene itself [35,36]. The aberrant expression profiles of FLG, FLG2, LOR, and IVL may be corrected with biological therapies or topical corticosteroids, particularly by inhibiting IL-4 and IL-13 in the context of AD [37,38]. Collectively, our findings suggest that POL primarily inhibits the JAK1/STAT3 signaling cascade; diminishes Th2-type inflammatory cytokines such as IL-4, IL-13, and IL-31; and restores the expression of skin barrier-associated genes, including FLG and LOR, thereby attenuating Th2 inflammation and facilitating the restoration of skin barrier integrity.

In this investigation, we conducted a comprehensive metabolomic analysis of fecal samples from mice with AD to elucidate the metabolic mechanisms underlying the therapeutic efficacy of POL in AD. A total of 82 intersecting differentially abundant metabolites were discerned, thereby revealing the distinctive metabolic profile associated with POL intervention in AD. The identified metabolites were predominantly fatty acyls and steroid lipids, which are chiefly involved in the biosynthesis of unsaturated fatty acids and steroid hormones. Notably, long-chain fatty acids (C14–C18/C20), particularly stearic acid (C18), are significantly elevated in AD, along with elongation aberrations of very long-chain (C20–C26) and ultralong-chain (>C26) fatty acids [39]. This decrease in lipid chain length correlates with reduced lipid packing density and compromised skin barrier integrity [40]. Our findings showed that stearic acid concentrations were markedly increased in AD mice, whereas POL administration significantly reduced these levels, thereby facilitating the restoration of skin barrier function. Additionally, POL administration restored the diminished levels of several unsaturated fatty acids, including (±)18-HEPE, docosahexaenoyl ethanolamide, (±)12(13)-DiHOME, and (±)9(10)-DiHOME.

ω-3 PUFAs are extensively recognized for their anti-inflammatory properties, primarily attributed to their capacity to inhibit the synthesis of arachidonic acid-derived prostaglandins and leukotrienes [41,42]. Notably, ω-3 PUFAs, including EPA and DHA, are significantly diminished in the dermal and serum profiles of patients with AD [20]. (±)18-HEPE, a biochemical derivative of EPA, strongly inhibits neutrophil chemotaxis and provokes robust anti-inflammatory responses [43]. Docosahexaenoyl ethanolamide, an endogenous human metabolite of DHA, impedes mast cell degranulation and modulates allergy-associated immune cells, thereby mitigating IgE-mediated allergic reactions [44]. Simopoulos and Omara-Alwala et al. reported that POL is a rich source of ω-3 PUFAs, including EPA, DHA, and docosapentaenoic acid (DPA) [45,46]. In line with this, the abundant ω-3 PUFA signals detected in the POL extract correlated with elevated levels of downstream metabolites, including (±)18-HEPE and docosahexaenoyl ethanolamide, both of which possess well-documented anti-inflammatory and antiallergic properties. This observation suggests that the ω-3 PUFA constituents of POL may contribute, at least in part, to its therapeutic effects in AD. Moreover, POL is also a source of ω-6 fatty acids, including linoleic acid. Our investigational study revealed that POL administration increased levels of downregulated linoleic acid metabolites, such as (±)12 (13)-DiHOME and (±)9(10)-DiHOME, further corroborating its therapeutic efficacy in AD.

DHEA is an endogenous steroid hormone that plays a pivotal role in steroid hormone biosynthesis. It has the potential to mitigate inflammatory responses, and serum levels of DHEA are significantly diminished in individuals afflicted with immune-inflammatory disorders, including AD and asthma [47]. Our study similarly observed decreased DHEA levels in AD mice. DHEA facilitates Th1 cell differentiation and helps maintain a homeostatic balance between Th1 and Th2 functions [48,49]. Empirical evidence suggests that it ameliorates skin inflammation and reduces eosinophil and mast cell infiltration in AD mice by attenuating Th2 cytokine levels [50]. In our study, POL treatment restored attenuated DHEA levels in AD mice, underscoring its ability to elevate DHEA levels, which may help mitigate Th2-type inflammatory responses. Intriguingly, Zhang et al. reported that dysregulated steroid hormone synthesis, promoted by Th2 cytokines, contributes to disrupted skin barrier integrity in AD [51]. DHEA functions as a circulating precursor to steroid hormones and serves as a substrate for 3β-hydroxysteroid dehydrogenase 1 (HSD3B1), a critical rate-limiting enzyme that governs steroid hormone biosynthesis [52]. Upregulation of HSD3B1, which is induced by IL-4 and IL-13, promotes the conversion of DHEA into androgens such as androstenedione and testosterone [53]. Diminished DHEA levels have been correlated with dysfunction of the skin barrier and lipid abnormalities in patients with AD [51]. Dupilumab, a monoclonal antibody that targets the shared alpha subunit of the IL-4 and IL-13 receptors (IL-4Rα), has been shown to downregulate HSD3B1 expression, curtail DHEA consumption, and restore both skin barrier function and lipid metabolism in individuals suffering from AD [51,54,55]. Strategic modulation of steroid hormone biosynthesis represents a promising therapeutic strategy for restoring skin barrier integrity. Our investigation revealed that POL can modulate steroid hormone biosynthesis pathways and restore diminished DHEA levels, which is one mechanism through which it facilitates the restoration of skin barrier function.

## 5. Conclusions

In summary, POL treatment effectively attenuates DNFB-induced AD-like characteristics in C57BL/6 mice, concurrent with suppression of the Th2 inflammatory cascade and restoration of epidermal barrier integrity. Moreover, POL demonstrated its therapeutic effect by modulating the biosynthesis of unsaturated fatty acids and steroid hormones, thereby influencing the pathways described above. These findings contribute to the pharmacological understanding of POL and lay the groundwork for its prospective clinical application in the management of AD, pending further validation across sexes, diverse AD models, and standardized preparations. Several limitations warrant consideration. First, the observed parallel between restored lipid profiles and suppressed JAK/STAT phosphorylation does not establish a causal relationship, as no direct interventions (e.g., receptor agonists/antagonists or genetic manipulations) were applied to validate this linkage. Thus, our interpretation regarding the interplay between lipid metabolism and JAK/STAT signaling remains correlational. Second, the exclusive use of male C57BL/6 mice and a single DNFB-induced AD model constrains the generalizability of our findings across sexes, genetic backgrounds, or alternative sensitization protocols. Third, because the POL aqueous extract is a complex phytochemical mixture, we have yet to quantitatively standardize its bioactive constituents—an essential prerequisite for batch consistency and eventual clinical translation. These limitations underscore the need for further mechanistic dissection, including systematic fractionation to identify active principle(s) and orthogonal in vitro/in vivo validation of the functional roles of specific lipid mediators in JAK/STAT modulation.

## Figures and Tables

**Table 1:** Primer sequences for RT–PCR.

Gene	Primer Sequence
*IL4-F*	5′-GCACGGAGATGGATGTG-3′
*IL4-R*	5′-CAAGCATGGAGTTTTCCC-3′
*IL-13F*	5′-GAAGCAGTGGGCTCTGG-3′
*IL-13R*	5′-CGTGGCAGACAGGAGTG-3′
*IL-31F*	5′-TCCAGAGACCACAGGCA-3′
*IL-31R*	5′-TACAGCAAAGCAGCACCA-3′
*LOR-F*	5′-CTGTGGCGGCGGCTACTC-3′
*LOR-R*	5′-GCTCTGGCACTGATACTGTTGG-3′
*FLG-F*	5′-GCAATCCCACTCCAAACCATCTC-3′
*FLG-R*	5′-GACTGTCCTCTTCCTCCTGATCC-3′
*actin-F*	5′-CTATTGGCAACGAGCGGTTCC-3′
*actin-R*	5′-GCACTGTGTTGGCATAGAGGTC-3′

**Table 2:** Antibodies used for Western blotting.

Antibody	Catalog No.	Supplier
JAK1	310108	ZEN BIO
Phospho-JAK1 (Tyr1034/1035)	74129T	Cell Signaling Technology
STAT1	R25801	ZEN BIO
Phospho-STAT1 (Tyr701)	340797	ZEN BIO
STAT3	ZH5030	Yipin Zhonghe
Phospho-STAT3 (Tyr705)	ZH5095	Yipin Zhonghe
β-Actin	66009-1-Ig	Proteintech
Goat Anti-Rabbit IgG H&L (HRP)	511203-100ul	ZEN BIO

**Table 3:** Main chemical constituents identified in the aqueous extract of POL.

Chemical Compound Name	Formula	Class I	PubChem CID	RT (Min)
FFA(18:0)	C_18_H_36_O_2_	Lipids	5281	7.395
(2-oxo-2,3-dihydro-1H-indol-3-yl)acetic acid	C_10_H_9_NO_3_	Alkaloids	3080590	3.4572
1-Linoleoyl-sn-glycero-3-phosphocholine	C_26_H_51_NO_7_P^+^	GP	5280646	6.788
6-Methylcoumarin	C_10_H_8_O_2_	Lignans and Coumarins	7092	1.9109
Vidarabine	C_10_H_13_N_5_O_4_	Nucleotides and derivatives	21704	2.1112
Citric acid	C_6_H_8_O_7_	Organic acids	311	0.8123
Polydextrose	C_12_H_22_O_11_	Others	872	0.7991
Ethyl alpha-linolenate	C_20_H_34_O_2_	Lipids	5367460	6.8275
Usnic acid	C_18_H_16_O_7_	Phenolic acids	5646	6.3669
2-Amino-1-phenylethanol	C_8_H_11_NO	Alkaloids	1000	2.1508
4-Hydroxy-3-methoxycinnamaldehyde	C_10_H_10_O_3_	Phenolic acids	5280536	3.5495
Ferulic acid	C_10_H_10_O_4_	Phenolic acids	445858	4.2242
(2E,4E)-11-Methoxy-3,7,11-trimethyldodeca-2,4-dienoic acid	C_16_H_28_O_3_	Terpenoids	5353760	6.166
PA(22:1(13Z)/22:6(4Z,7Z,10Z,13Z,16Z,19Z))	C_47_H_79_O_8_P	GP	131822087	7.5685
2′-Hydroxy-4,4′,6′-trimethoxychalcone	C_18_H_18_O_5_	Flavonoids	5355469	6.2664
Dihydrodaidzein	C_15_H_12_O_4_	Flavonoids	176907	1.7115
(R)-Higenamine	C_16_H_17_NO_3_	Alkaloids	114840	2.8597
Obamegine	C_36_H_38_N_2_O_6_	Lignans and Coumarins	441064	6.6065
Artemetin	C_20_H_20_O_8_	Flavonoids	5320351	4.6796
Schisandrin B	C_23_H_28_O_6_	Lignans and Coumarins	108130	7.3653
Aleuritic acid	C_16_H_32_O_5_	Lipids	10790	5.599
Niacinamide	C_6_H_6_N_2_O	Others	936	1.672
Eicosa-8,11,14-trien-5-ynoic acid	C_20_H_30_O_2_	Lipids	6443815	5.1358
17-Hydroxylinolenic acid	C_18_H_30_O_3_	Lipids	10708957	7.0481
Caffeic acid	C_9_H_8_O_4_	Phenolic acids	689043	3.7386
Thiamine	C_12_H_17_N_4_OS^+^	Others	1130	1.3948
N-[2-(3,4-Dihydroxyphenyl)ethyl]hexadecanamide	C_24_H_41_NO_3_	Lipids	16759163	3.4076
Chlorogenic Acid	C_16_H_18_O_9_	Phenolic acids	1794427	3.1768
Quercetin 3-xylosyl-(1->6)-glucoside	C_26_H_28_O_16_	Flavonoids	44259141	2.3573
Dopamine	C_8_H_11_NO_2_	Alkaloids	681	1.2563
Ascorbyl palmitate	C_22_H_38_O_7_	Others	54680660	8.7674
Arachidonic acid	C_20_H_32_O_2_	Lipids	444899	6.5875
Eicosapentaenoic acid	C_20_H_30_O_2_	Lipids	446284	6.4069
Syringaresinol	C_22_H_26_O_8_	Lignans and Coumarins	100067	5.4184
Luteolin-7-o-glucuronide	C_21_H_18_O_12_	Flavonoids	13607752	4.444
Linoelaidic acid	C_18_H_32_O_2_	Lipids	5282457	6.5677
gamma-Linolenic Acid	C_18_H_30_O_2_	Lipids	5280933	8.1056

Note: LC-MS/MS-identified compounds. Identification criteria included retention time matching, accurate mass (<5 ppm), and MS/MS fragmentation patterns, which were cross-referenced with the HMDB and METLIN databases. The internal standard used was 2-chloro-L-phenylalanine. For detailed LC-MS conditions, please refer to Section 2.2.

**Table 4:** Differentially expressed metabolites.

	N vs. M			POL vs. M		
Metabolite	FC	*p*	Reg.	FC	*p*	Reg.
Stearic acid	0.667	0.031	↓	0.509	0.017	↓
Acetylcarnitine	0.604	0.008	↓	0.623	0.013	↓
Isobutyryl carnitine	0.759	0.013	↓	0.709	0.003	↓
5-Phenylvaleric Acid	0.413	0.000	↓	0.608	0.009	↓
(±)18-HEPE	2.338	0.000	↑	1.483	0.044	↑
Avocadyne 1-acetate	2.165	0.001	↑	1.591	0.016	↑
13-Hpotre(R)	2.493	0.000	↑	2.476	0.000	↑
acetoacetate	1.673	0.000	↑	2.173	0.000	↑
Sebacic acid	1.39	0.045	↑	2.296	0.001	↑
(±)12(13)-DiHOME	2.193	0.015	↑	3.825	0.008	↑
(±)9(10)-DiHOME	1.549	0.028	↑	2.962	0.008	↑
Prostaglandin E1	0.673	0.009	↓	0.452	0.000	↓
Anandamide	1.844	0.021	↑	1.551	0.019	↑
16,16-Dimethyl prostaglandin A2	2.609	0.001	↑	1.996	0.003	↑
Prostaglandin B2	0.777	0.016	↓	1.382	0.042	↑
Prostaglandin E2-1-glyceryl ester	1.745	0.026	↑	2.936	0.038	↑
Docosahexaenoyl Ethanolamide	5.296	0.000	↑	1.794	0.005	↑
Dehydroepiandrosterone	2.762	0.000	↑	2.277	0.005	↑
23-Nordeoxycholic acid	0.357	0.025	↓	0.314	0.017	↓
Dehydrocholic acid	0.602	0.000	↓	0.567	0.000	↓
All-Trans-13,14-Dihydroretinol	0.541	0.031	↓	0.555	0.039	↓
Lysopc 17:0	4.027	0.011	↑	2.312	0.039	↑
3-Hydroxypicolinic acid	0.585	0.024	↓	0.487	0.006	↓
Norfloxacin	1.473	0.012	↑	1.819	0.000	↑
Enrofloxacin	0.643	0.047	↓	1.965	0.029	↑
4-Hydroxybenzoic acid	1.819	0.000	↑	2.089	0.001	↑
Naratriptan	0.169	0.047	↓	0.148	0.046	↓
Phenylglyoxylic acid	0.426	0.008	↓	0.449	0.011	↓
5-Methyl-dl-tryptophan	1.897	0.000	↑	1.294	0.018	↑
N-Acetyl-L-carnosine	2.641	0.000	↑	1.547	0.044	↑
Anserine	1.788	0.013	↑	1.423	0.017	↑
Homogentisic Acid	2.245	0.039	↑	1.697	0.016	↑
Tryptophanol	1.921	0.026	↑	2.01	0.024	↑
Ne-(1-Carboxymethyl)-L-lysine	0.543	0.037	↓	2.163	0.045	↑
Boc-beta-cyano-L-alanine	1.729	0.023	↑	2.133	0.002	↑
trans-Cinnamaldehyde	0.713	0.040	↓	0.503	0.002	↓
NSI-189	0.593	0.004	↓	0.713	0.008	↓
1,3-Dimethyluric Acid	1.988	0.008	↑	1.684	0.033	↑
Quinoline-4-carboxylic acid	1.432	0.005	↑	1.286	0.049	↑
N-Desmethyltramadol	13.53	0.002	↑	3.724	0.008	↑
Veratramine	2.029	0.040	↑	3.057	0.001	↑
N-Acetyl-5-aminosalicylic acid	1.689	0.047	↑	2.019	0.010	↑
L-Dopa	2.001	0.014	↑	2.289	0.004	↑
Neopterin	7.499	0.000	↑	2.878	0.000	↑

Note: Using the Model group as the control, changes in the Normal and POL-M groups were analyzed. FC represents the fold change of metabolites; p is the *p*-value from the *t*-test; Reg. indicates regulation of metabolites compared with the Model group. FC < 1 indicates metabolite downregulation (↓), while FC > 1 indicates metabolite upregulation (↑).

## Data Availability

The data that support the findings of this study are available from the corresponding author upon request.
